# Transformation and Overexpression of Primary Cell Wall Synthesis-Related Zinc Finger Gene *Gh_A07G1537* to Improve Fiber Length in Cotton

**DOI:** 10.3389/fpls.2021.777794

**Published:** 2021-11-05

**Authors:** Abdul Razzaq, Muhammad Mubashar Zafar, Pengtao Li, Ge Qun, Xiaoying Deng, Arfan Ali, Abdul Hafeez, Muhammad Irfan, Aiying Liu, Maozhi Ren, Haihong Shang, Yuzhen Shi, Wankui Gong, Youlu Yuan

**Affiliations:** ^1^State Key Laboratory of Cotton Biology, Key Laboratory of Biological and Genetic Breeding of Cotton, The Ministry of Agriculture, Institute of Cotton Research, Chinese Academy of Agricultural Science, Anyang, China; ^2^Institute of Molecular Biology and Biotechnology, The University of Lahore, Lahore, Pakistan; ^3^School of Biotechnology and Food Engineering, Anyang Institute of Technology, Anyang, China; ^4^FB Genetics, Four Brothers Group, Lahore, Pakistan; ^5^Department of Biological Sciences, Forman Christian College, A Chartered University, Lahore, Pakistan; ^6^School of Agricultural Sciences, Zhengzhou University, Zhengzhou, China

**Keywords:** CSSLs, fiber length, zinc finger, genetic transformation, cotton biology

## Abstract

Molecular interventions have helped to explore the genes involved in fiber length, fiber strength, and other quality parameters with improved characteristics, particularly in cotton. The current study is an extension and functional validation of previous findings that *Gh_A07G1537* influences fiber length in cotton using a chromosomal segment substitution line MBI7747 through RNA-seq data. The recombinant *Gh_A07G1537* derived from the MBI7747 line was over-expressed in CCRI24, a genotype with a low profile of fiber quality parameters. Putative transformants were selected on MS medium containing hygromycin (25mg/ml), acclimatized, and shifted to a greenhouse for further growth and proliferation. Transgene integration was validated through PCR and Southern Blot analysis. Stable integration of the transgene (Δ*Gh_*A07G1537) was validated by tracking its expression in different generations (T_0_, T_1_, and T_2_) of transformed cotton plants. It was found to be 2.97-, 2.86-, and 2.92-folds higher expression in T_0_, T_1_, and T_2_ plants, respectively, of transgenic compared with non-transgenic cotton plants. Fiber quality parameters were also observed to be improved in the engineered cotton line. Genetic modifications of Gh*_A07G1537* support the improvement in fiber quality parameters and should be appreciated for the textile industry.

## Introduction

Cotton is one of the most important sources of natural cellulose in the world. The cotton boll protects seeds and delicate fibers (Von Mark and Dierig, 2014). This crop provides an excellent system for studying polyploidization and cell elongation (Wang et al., 2019) with more than 50 species which are further divided into eight diploid genomic groups (A–G, and K) and one tetraploid genomic group (AD; Wu et al., 2018). The growth of the textile industry is solely dependent on cotton crop production in more than 55 countries all over the globe (Kanat et al., 2018).

Zinc finger protein (ZFP) is one of the most significant and large families in plants which is characterized by the zinc finger motifs (Znf). The ZFPs are classified into 14 gene families, among which RING finger, LIM, DOF, AP2/EREBP, and WRKY have been reported to play a vital role in plant growth and development (Liu and Zhang, 2017). The ZFPs are categorized into 10 groups (C2H2, C2HC, C2HC5, C2C2, C3H, C3HC4, C4, C4HC3, C6, and C8) based on the number of cysteine and histidine residues and amino acids (Moore and Ullman, 2003). The cysteines and/or histidines coordinate with zinc ions to form a peptide structure (Hall, 2005). They have been involved in core biological processes like morphogenesis, signal transduction, development, and survival under environmental stresses (Stege et al., 2002). Among ZFPs, Cysteine3 Histidine (C3H) consists of three cysteines and one histidine coordinated by a zinc cation, which is reported as DNA/RNA binding proteins (Wang et al., 2008). Furthermore, CCCH genes play a vital role in hormone-regulated stress responses and cell fate determination. The Znf-CCCH has been reported to be involved in various developmental processes and adaptation under stress conditions. For example, *AtPEI1* of the CCCH gene in *Arabidopsis* is essential for heart-stage embryo formation (Li and Thomas, 1998). Overexpression of the CCCH gene, *OsDOS*, caused a delay in leaf senescence in rice by affecting the jasmonic acid pathway (Kong et al., 2006). Similarly, overexpression of the CCCH gene (*GhZFP1*) in cotton resulted in enhanced salt tolerance and disease resistance (Guo et al., 2009). Hence, numerous studies confirm the involvement of ZFPs in the fiber quality of cotton.

Many factors influence the cotton fiber quality (Xiao et al., 2019) and several efforts have been made to improve its quality and yield (Ahmed et al., 2018a). The cotton fiber is an epidermal single-cell extension, which consists of four overlapping and sequential stages of differentiation: initiation, elongation, secondary wall synthesis, and maturation (Ahmed et al., 2018a). Its development is controlled by numerous genes, transcription factors, and phytohormones (Xiao et al., 2019). The qualitative and quantitative traits of the cotton fiber are significantly regulated by the genes involved in cell wall synthesis and extension (Guo et al., 2016). Several genes and transcription factors for expansin (Bajwa et al., 2015), cellulose synthase (Arioli et al., 1998), sucrose synthase (Ahmed et al., 2020), and actin have been transformed successfully into cotton for the improvement of fiber quality and yield (Ahmed et al., 2018a).

The molecular mechanisms involved in ovule epidermal cell development may help to explore cotton fiber quality parameters (Pu et al., 2008) which can potentially have an impact on fiber length, fiber strength, micronaire value, and maturity (Seagull et al., 2000). The molecular genetics of the genes and their isoforms provide a better understanding of their function at specific fiber developmental stages (Manik et al., 2009). Several differentially expressed genes are required at different fiber development stages (Yang et al., 2009). However, fewer of the genes regulate the biosynthesis of fiber-specific structural proteins, enzymes, waxes, and polysaccharides (Li et al., 2002) to improve the cotton fiber quality (Rapp et al., 2010).

Chromosome segment substitution lines (CSSLs) are permanent populations possessing the same genetic background as a recurrent parent with a difference of one or few introgressed chromosomal segments. It effectively eliminates the interference of the genetic background, of which permanent populations are also used to determine the QTLs with minor effects. Therefore, CSSLs are considered to be the ideal material for QTL fine mapping, investigation of QTL interaction, and gene cloning. Since the construction of CSSLs by Eshed and Zamir (1994), these have successfully been applied in rice, corn, and other plants (Liu et al., 2015). However, there was a low frequency of the CSSLs conducted in cotton for QTL studies. It has been reported that 17 CSSLs of Sea iceland cotton (*Gossypium barbadense*) in TM-1 background of *Gossypium hirsutum* were constructed (Stelly et al., 2005). Furthermore, it was reported by the same research group that sea island cotton has a significant contribution to fiber quality traits influenced by multiple genes (Zhang et al., 2014).

At our ICR-CAAS, three CSSLs (MBI7561, MBI7285, and MBI7747) were developed from CCRI45 (*G. hirsutum*) and Hai1 (*G. barbadense*) using the conventional breeding methods and modern molecular marker techniques (Shi et al., 2015), which subsequently were subjected to transcriptome sequencing together with their parents. Several differentially expressed genes (DEGs) responsible for fiber length were identified and suggested for further functional studies in *Arabidopsis* and subsequently in cotton (Li et al., 2021). The current study is a continuation of the aforementioned hypothesis, suggesting that the differentially expressed gene (*Gh_A07G1537*) is located on chromosome 7 and it belongs to the CCC-H zinc finger gene superfamily which regulates the primary cell wall synthesis. Hence, overexpression of the afore-mentioned gene has a great potential to improve fiber length in cotton.

## Materials and Methods

### Collection of Material

The cotton bolls of CCRI45, Hai1, and MBI7747 at 20 DPA were collected from the field of ICR-CAAS and brought to the laboratory in liquid nitrogen. The fiber was extracted from the collected cotton bolls and stored at −80°C.

### Isolation of RNA and cDNA Synthesis

The total RNAs of all three materials (CCRI45, Hai1, and MBI7747) were extracted from the cotton fiber of 20 DPA using the RNAprep pure plant kit (Tiangen, Beijing, China) method. The genomic DNA contaminations were removed by DNase1. The quantity and integrity of isolated RNA were measured by Nano-Drop 2000 spectrophotometer (Thermo-Scientific, United States) and visualized on a 1% agarose gel electrophoresis. The quantified RNA of the materials was subjected to prepare cDNA transcript in a reaction volume of 20μl and final dilution was prepared in 100μl using PrimeScript® RT Reagent Kit (Perfect Real Time, Takara Biotechnology Co., Ltd., Dalian, China).

### Detection of *Gh_A07G1537* Gene in CCRI45, Hai1, and MBI7747

**Figure 1:**
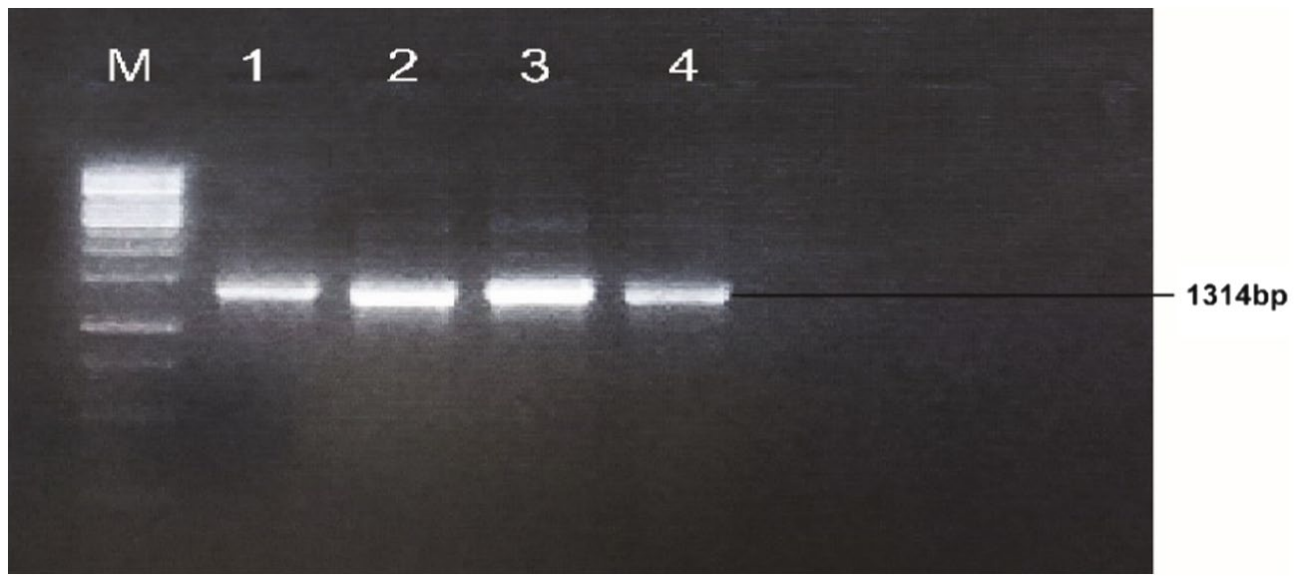
Identification and isolation of *Gh_A07G1537* gene from CCRI45, Hai1 and MBI7747; Lane M: 1kb DNA ladder; Lane 1: amplification from cDNA of CCRI45; Lane 2: amplification from Hai1; Lanes 3 and 4: amplification from the cDNA of genotype MBI7747.

A gene was amplified from the cDNA template with optimal PCR conditions; initial denaturation temperature was 95°C for 3min, denaturation 95°C for 45s, annealing at 65.4°C for 45s, extension 72°C for 2min and final extension was 72°C for 10min. The total reaction volume was prepared in 25μl; cDNA template 1μl, F-primers 1μl, R-reverse primer 1μl, master mix Hi-Fi 12.5μl and water 9.5μl. An agarose gel of 1% was used in a freshly prepared 1X TAE buffer to visualize the bands of PCR products (see Figure 1). The gene-specific primer sequences; Forward primer sequence: 5'CCATGGATGCCTGATAATCGGCAAGTTCAGAAC3' and Reverse primer sequence: 5'AGATCTTCAATCATCATGTGAGGTTTTCGAAGAACCC 3' were used for PCR detection.

### Cloning and Sequencing of the Positive Clones

**Figure 2:**
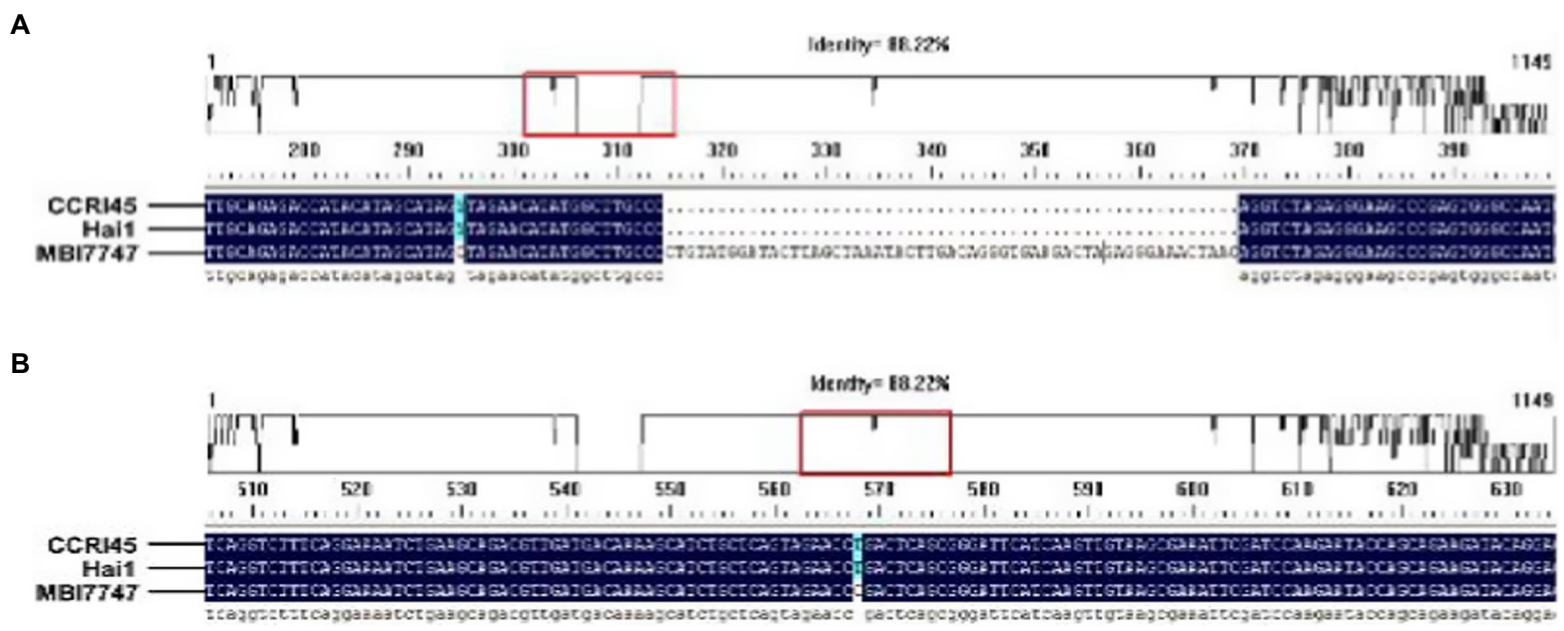
Analysis of sequencing results showing different SNPs and Indel in MBI7747 (*Gh_A07G1537*) as compared with its parents of CCRI45 and Hai1; **(A)** detection of Indel of 55bp and **(B)** detection of SNPs.

A 4μl PCR purified product and 1μl blunt zero cloning vector (TOPO TA cloning, cat#45-0641, Invitrogen) were taken and incubated at 27°C for 10min using a thermal cycler. A reaction volume of 5μl was transferred into a 1.5ml Eppendorf tube containing 50μl competent cells. The Eppendorf tube was then incubated in ice for 30min. The tube was taken out and exposed to heat shock for 1min at 42°C using a water bath. Then the tube was immediately removed and put back into the ice for a quick chill for 2–3min. An aliquot (450μl) of LB (tryptone 10g/L, yeast 5g/L, NaCl 5g/L) was added to the tube and incubated on a shaker at 37°C for 55min with 200rpm. Then a 25–100μl of the cells in LB were spread over the Kanamycin (50mg/L) plates and incubated at 37°C for at least 12h or overnight. After overnight incubation, discrete colonies of each material (CCRI45, Hai1, MBI7747) were picked up into 5ml of the LB medium containing Kanamycin (50mg/L) and incubated at 37°C for overnight for PCR amplification. The plasmids of positive clones were isolated using a plasmid isolation kit (Thermo-Scientific, Cat#K0503). The positives plasmids were sequenced (Sangon biotech Shanghai, China) and analyzed using the DNAMAN software version 9 (see Figure 2).

### Ligation and Transformation of the Gene Into *Agrobacterium tumefaciens*

**Figure 3:**
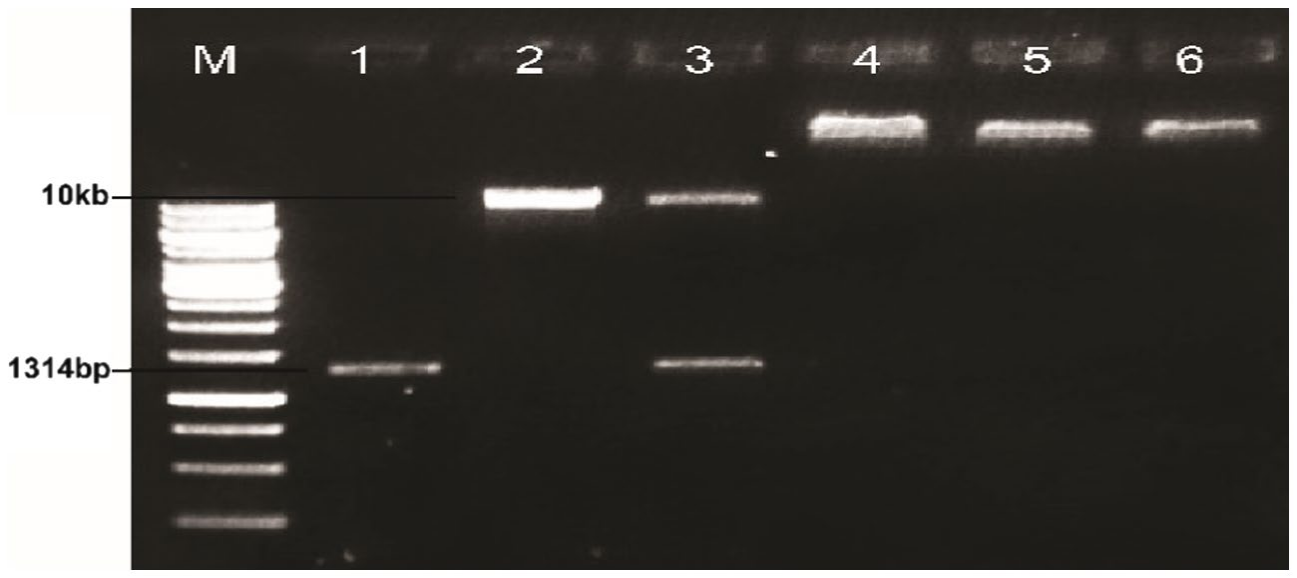
Ligation of Δ*Gh_A07G1537* gene into PCAMBIA2300; Lane M: 1kb molecular weight marker; Lane 1: *Gh_A07G1537*; Lane 2: PCAMBIA2300; Lane 3: non-ligated mixture without ligase; Lanes 4–6: ligation of MBI7747 (*Gh_A07G1537*).

The plasmid of the positive clone and plant expression vector PCAMBIA2300 were digested with the restriction enzymes *BglII* and *NcoI* and incubated at 37°C for 2h. The purified products were then ligated using a fast ligation kit (Thermo-Scientific, K1423.) at 22°C for 15min. The ligated products were then run on the 1% agarose gel electrophoresis (see Figure 3). An aliquot of 5μl of the purified product was transferred into 50μl competent cells of *Agrobacterium tumefaciens* LBA4404 through the liquid nitrogen method. A volume of 450μl of YEP (peptone 10g/L, yeast extract 10g/L, NaCl 5g/L) was added to each tube and incubated on a shaker at 28°C for 48h at 200rpm. A 25–100μl of the cells in YEP were spread over the plates containing Kanamycin (50mg/L) and rifampicin (50mg/L) and incubated at 28°C for 48h.

### Confirmation of Gene Constructs in *A. tumefaciens* Through PCR

**Figure 4:**
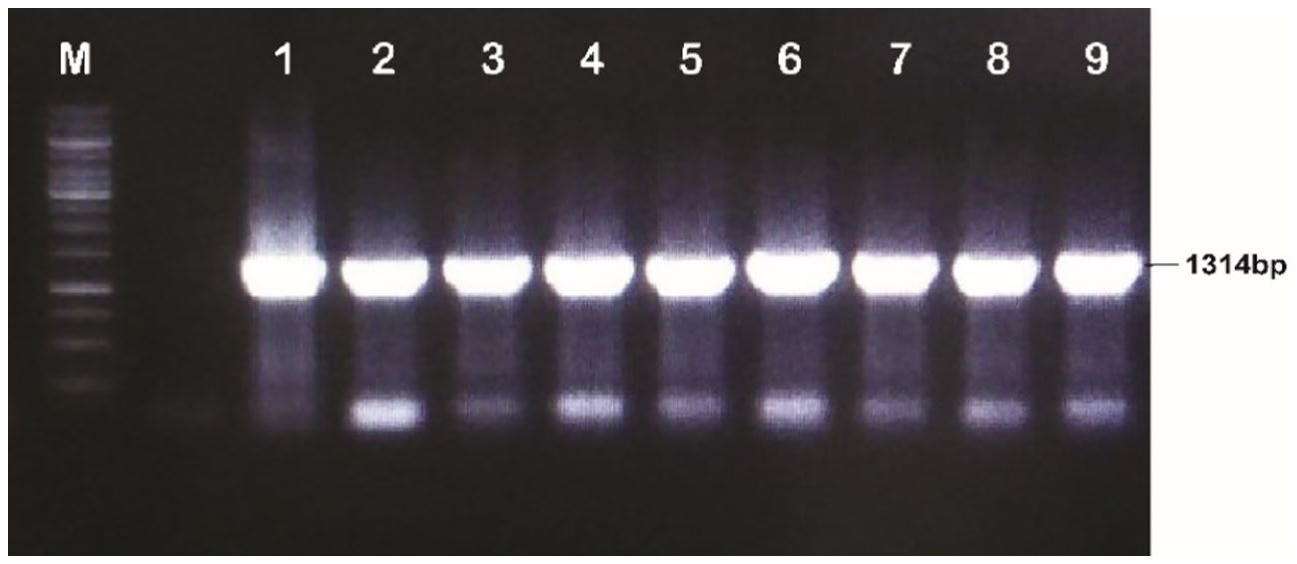
Confirmation of recombinant *Agrobacterium tumefaciens* through PCR (Full length primers); Lane M: 1kb molecular weight marker; Lanes 1–9: MBI7747 (*Gh_A07G1537*).

**Figure 5:**
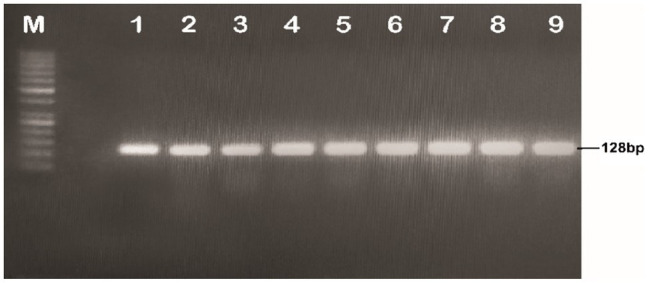
Confirmation of the recombinant *A. tumefaciens* through PCR (Short length primers); Lane M: 1kb molecular weight marker; Lanes 1–9: MBI7747 (*Gh_A07G1537*).

The discrete and isolated colonies of *A. tumefaciens* that appeared on the YEP plates were picked and cultured in 5ml of the YEP medium and incubated at 28°C for 48h. A volume of 100μl of the culture was centrifuged at 14,000rpm for 10min. The supernatant was discarded and resuspended in the pellet in 50μl of 1X TE buffer. The suspension was shifted to PCR tubes and incubated at 98°C for 12min. The tubes were slightly spun and a volume of 5μl of the clear supernatant was taken as a PCR template. The gene in *A. tumefaciens* was detected with full-length primers using PCR conditions; initial denaturation temperature was 95°C for 3min, denaturation 95°C for 45s, annealing 65.4°C for 45s, extension 72°C for 2min and final extension at 72°C for 10min, whereas detection through short length primers using PCR conditions was done at the following conditions; initial denaturation temperature was 95°C for 3min, denaturation 95°C for 45s, annealing 60°C for 45s, extension at 72°C for 2min and final extension at 72°C for 10min. The total reaction volume was prepared in 25μl; cDNA template 1μl, F-primers 1μl, R-reverse primer 1μl, master mix Hi-Fi 12.5μl and water 9.5μl (see Figures 4, 5).

### Transformation of the Gene Into Cotton

**Figure 6:**
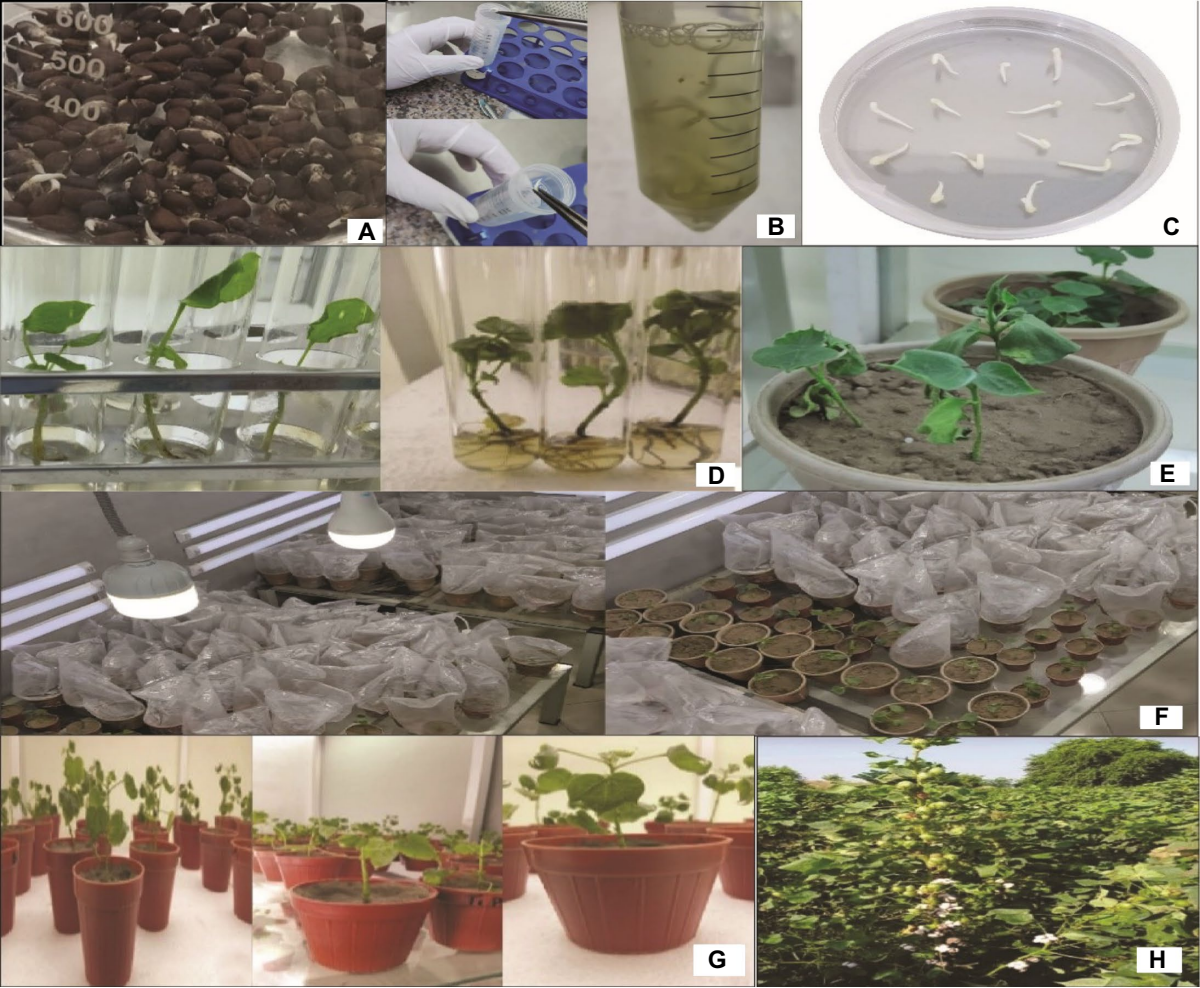
A schematic procedure of *Gh_A07G1537* gene transformation in cotton; **(A,B)** soaking of seeds, **(C)** shifting of embryos on MS plates, **(D)** shifting of embryos into the MS tubes, **(E–G)** shifting of plants into the pots, and **(H)** shifting of plants into the field.

The seeds of CCRI24 were delinted, surface-sterilized, and soaked at 30°C for 48h. The germinated seedlings were used for transformation using the shoot apex cut method (Ulian et al., 1988). The embryos, after injury, were inoculated with the selected transformant *A. tumefaciens* strains harboring the gene construct in the MS medium (4.4g/L, sucrose 30g/L, phytagel 2.4g/L) cultured for 1h at 28°C. The embryos were allowed to grow on the MS medium plates supplemented with cefotaxime (100mg/L) followed by screening in MS tubes supplemented with hygromycin (25mg/ml) for 6weeks. After screening, the cotton plants from the tubes were transplanted into pots containing an equal proportion of clay, peat moss, and sand (1:1:1). Subsequently, the putative transgenic cotton plants were transplanted in the greenhouse of Four Brothers Genetics Inc. for acclimatization and hardening followed by molecular analysis (see Tables 1–3 and Figure 6).

**Table 1:** Numerical data for transformation experiments.

Exp. No.	No. of embryos isolated	Agrobacterium treated embryos	Embryos on MS plates	Died	Selection tubes	Plantlets died	Plants transferred to pots	plants died in pots	Plants shifted to greenhouse
1	105	104	104	95	9	3	6	3	3
2	122	114	114	109	5	2	3	1	2
3	110	109	109	102	7	3	4	2	2
4	98	96	96	91	5	2	3	0	3
5	103	99	99	94	5	3	2	0	2
6	180	175	175	171	4	2	2	1	1
7	183	179	179	173	6	4	2	0	0
Total	901	876	876	835	41	19	22	7	13

**Table 2:** Germination index.

No. of petri plates	Total seeds	No. of germinated seeds	No. of ungerminated seeds	Germination index
1	30	19	11	63.33%
2	30	21		

**Table 3:** Transformation efficiency.

Agrobacterium treated embryos	Control plants	Plants shifted to greenhouse		Transformation efficiency	
		Control plants	Experimental	Control plants	Experimental
876	45	20	13	44.44%	1.48%

### Detection of the Gene in Putative Transgene Cotton Through PCR

**Figure 7:**
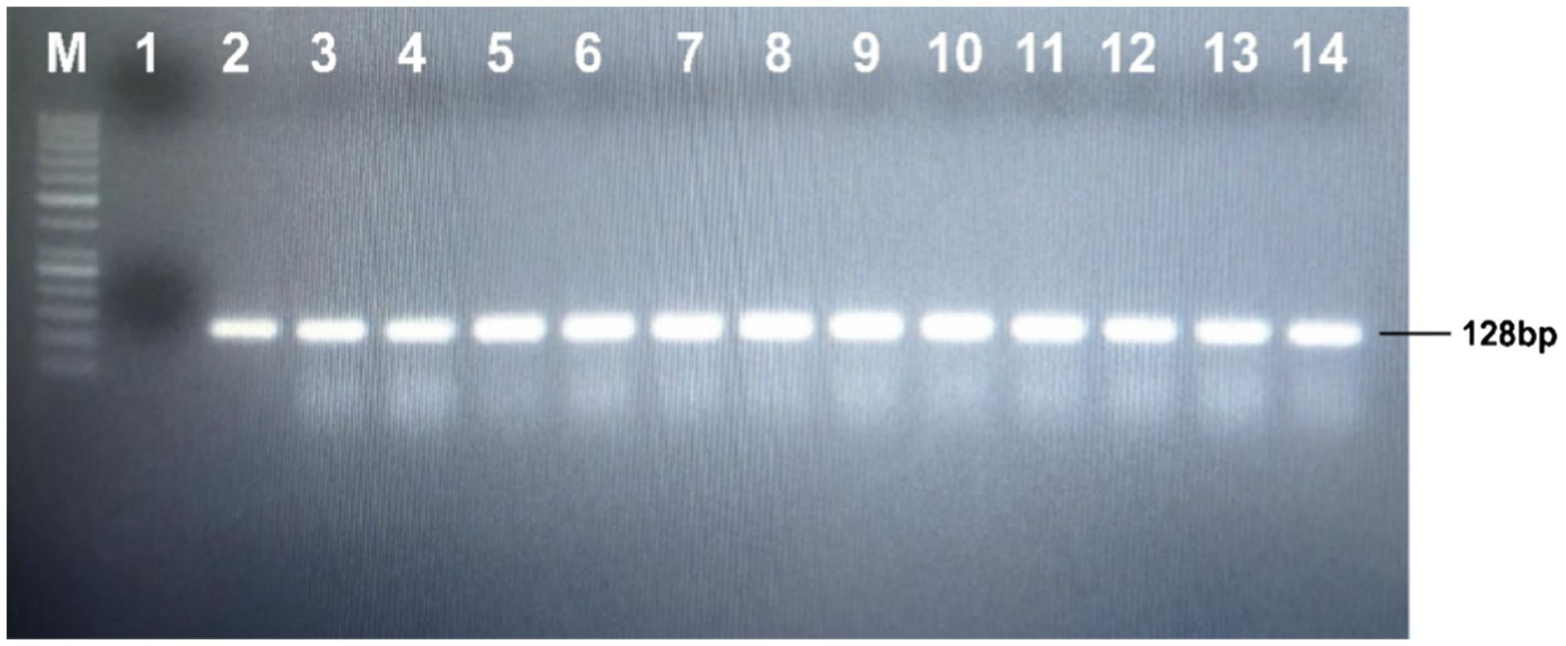
Detection of the transgene into putative transgenic cotton line through PCR; Lane M: 1kb molecular weight marker; Lane 1: negative control; Lane 2: positive control; Lanes 3–14: putative transgenic cotton plants.

The leaves of the putative transgenic cotton were taken for the confirmation of the gene (*Gh_A07G1537*), through PCR using the manufacturer protocol Green Plant direct PCR master mix kit, (Thermo-Scientific) using gene-specific short length primers; Forward primers 5' TTCTGCTGGTATTCTCGGATCG 3': Reverse primers 5' TGGGTTGATCAGGTCTTTCAGG 3' (see Figure 7).

### Detection of Stable Integration of a Gene in Putative Cotton

**Figure 8:**
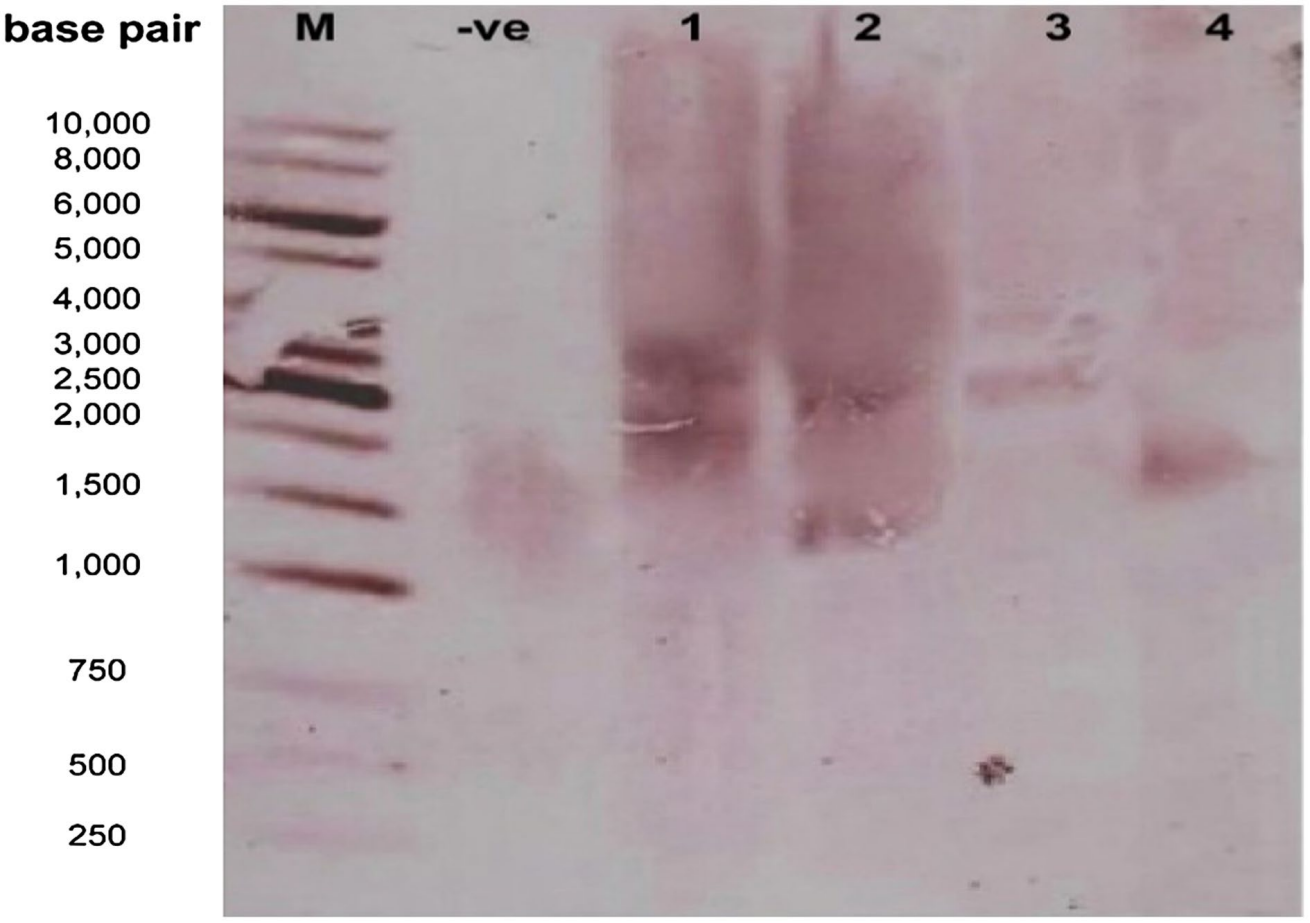
Confirmation of transgene integration into the cotton genome by the Southern Blot Analysis. Lane M: 1kb molecular weight marker; Lanes 1–3: transgenic cotton; Lane 4: non-transgenic.

The presence of a gene was confirmed through Southern blot analysis. The DNA of confirmed transgenic cotton was used to perform it. The extracted DNA was digested with *EcoRI* and a gene-specific probe was used for the detection of a gene (see Figure 8).

### RNA Extraction and cDNA Preparation

Primer pairs of *Gh_A07G1537* gene were designed using Primer3 Input Version 4.0. The RNA from the putative transgenic cotton was isolated from leaves using the Agilent kit (Agilent Technologies, Santa Clara, United States). The RNA was quantified in ng/μl using Nano-Drop 2000 spectrophotometer (Thermo-Scientific, United States) at 260 and 280nm. The DNase-treated total RNA was used to prepare cDNA using PrimeScript® RT Reagent Kit (Perfect Real Time, Takara Biotechnology Co., Ltd., Dalian, China) and cDNA was stored at −20°C.

### Expression Analysis of Cotton Transgene

**Figure 9:**
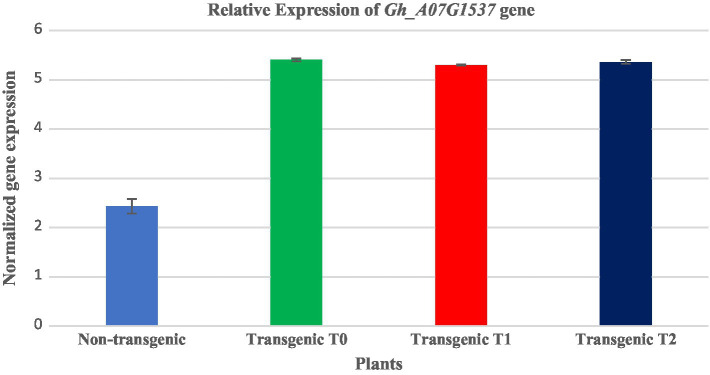
Comparative expression analysis of transgenic (T0, T1, and T2) and non-transgenic cotton plants three each through Realtime qRT-PCR. Expression of the transgene was higher in all three generations of transgenic cotton plants as compared with that in the non-transgenic plants. Blue color shows non-transgenic: green, red, and royal color show relative expression of the transgene in T0, T1, and T2 transgenic cotton plants, respectively.

The expression analysis of transgene cotton was performed by qRT-PCR using specific primers in triplicates with a product size of 128bp following the protocol of Maxima SYBR Green/ROX (Thermo-Scientific). The reaction mixture was prepared in a total of 20μl with the following components of 1μl of 10pmol of forward and reverse primers, 5μl of Maxima® SYBR Green/ROX qPCR Master Mix (2x) and 1μl (50ng/μl) of cDNA. The relative expression was determined and *GAPDH* primers were used as an internal control for normalization. All of the assays were performed in triplicate (see Figure 9).

### Analysis of Fiber Quality Parameters

**Figure 10:**
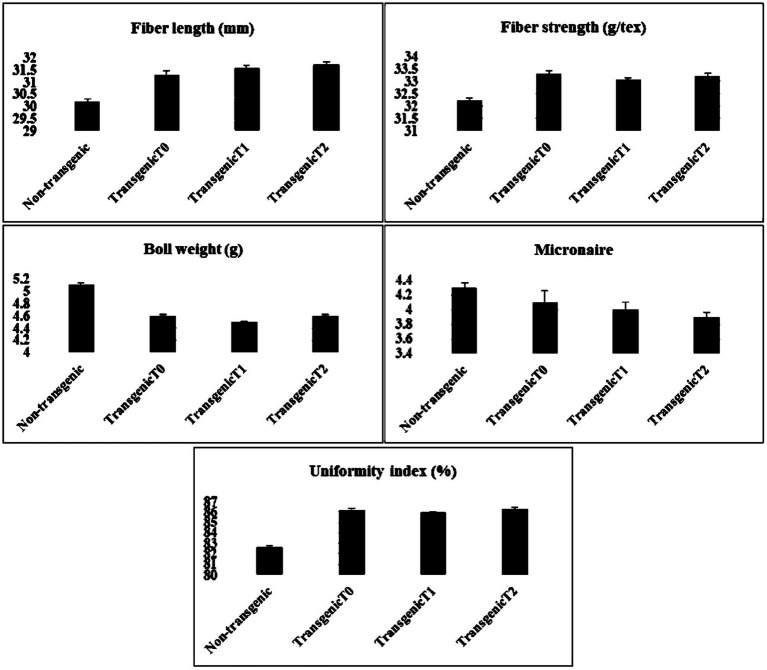
Comparative analysis of the fiber quality parameters in transgenic (T0, T1, and T2) and non-transgenic cotton plants three each. Fiber quality appeared to be improved owing to the expression of the transgene.

The careful examination of the expression of the gene in transgenic cotton revealed that the quality parameters such as fiber strength and length were of great interest. Fiber samples from the transgenic and non-transgenic cotton were collected and sent to the Central Cotton Research Institute (CCRI), Multan-Pakistan for the analysis of fiber quality parameters. The data were collected and evaluated for fiber quality parameters (see Figure 10).

## Results

### Isolation of Endogenous *Gh_A07G1537* Gene From Indigenous Cotton

The cotton bolls of the three cotton genotypes (CCRI45, Hai1, and MBI7747) at 20 DPA, were collected from the field and total mRNA was isolated for cDNA synthesis. The quality and integrity of the mRNA were checked using the Nano-Drop 2000 spectrophotometer (Thermo-Scientific, United States). The gene-specific primer sequences with forwarding primer sequence: 5'CCATGGATGCCTGATAATCGGCAAGTTCAGAAC 3' and reverse primer sequence: 5'AGATCTTCAATCATCATGTGAGGTTTTCGAAGAACCC 3' successfully amplified a fragment of 1,314bp (Figure 1).

### Cloning and Sequence Analysis of the Isolated *Gh_A07G1537* Gene

A PCR purified product and blunt zero cloning vector were taken and cloned in *Escherichia coli* competent cells. Plasmid DNA was isolated from the positive clones and was sequence characterized. The retrieved sequences were compared with the parents, to seek homology. It was found that the gene (*Gh_A07G1537*) isolated from the introgressed line was different in a few SNPs and Indel sequence of 55bp as compared with the gene sequences retrieved for its parent (Figure 2).

### Integration of the Δ*Gh_A07G1537* Into Plant Expression Vector PCAMBIA2300

The plasmid DNA of the positive clones and that of plant expression vector PCAMBIA2300 were digested with restriction endonucleases *BglII* and *NcoI*. Desired DNA fragments were eluted (Figure 3) and ligation was performed using the fast ligation kit (Thermo-Scientific, Cat#K1423). The ligation mixture was transformed into *E. coli* top 10 competent cells. After confirmation, it was transformed into *A. tumefaciens* using the liquid nitrogen method. The recombinant *Agrobacterium* cells were confirmed by the PCR using full-length gene primers and primers flanking internal sequences (short length). Amplification of a fragment of 1,314bp with full-length primer and a fragment of 128bp with short length primers confirmed the transformation of the gene construct into *Agrobacterium* (Figures 4, 5).

### Genetic Transformation of Cotton With Δ*Gh_A07G1537*

The seeds of the CCRI24 cotton variety were de-linted, surface sterilized, and soaked at 30°C for 48h. The germinated seedlings were used for the transformation of the Δ*Gh_A07G1537* gene using the shoot apex cut method. After initial selection and screening, total cellular DNA was isolated from the leaves of the putative cotton transformants (Figure 6). The isolated DNA was subjected to PCR to track transgene integration using gene-specific short-length primers. Amplification of a fragment of 128bp confirmed integration of Δ*Gh_A07G1537* into the cotton genome (Figure 7).

### Detection of Stable Integration of the Transgene Into the Cotton Genome

The presence of the transgene was also confirmed through the Southern Blot Analysis. The DNA of three confirmed transgenic cotton plants was extracted and digested with *EcoRI*, and a gene-specific probe was used for the detection of the transgene (Δ*Gh_A07G1537*) representing the size of 1,314bp. Blot analysis revealed that two copies of transgene were integrated into the host genome (lanes 1–3; Figure 8).

### Tracking Expression of the Transgene Δ*Gh_A07G1537* in Different Generations of Putative Transformants of Cotton

The expression analysis of three transgenic cotton plants was performed by qRT-PCR (Thermo-Scientific, United States) using gene-specific primers in triplicates following the protocol of Maxima SYBR Green/ROX (Thermo-Scientific, United States). The *GAPDH* gene was used as an internal control for the normalization of the reaction. The quantitative expression analysis was performed in T0, T1, and T2 plants of the putative transformants. The variations in the relative expression of the Δ*Gh_A07G1537* gene were observed in the three generations (Figure 9). The expression of the transgene (Δ*Gh_A07G1537*) was found to be 2.97-, 2.86-, and 2.92-folds higher in T0, T1, and T2 plants of transformed, respectively, as compared with that of the non-transgenic cotton plants.

### Analysis of Fiber Quality Parameters

After validation, integration, and overexpression of the transgene, fiber quality was also assessed of the transgenic cotton plants. Fiber samples were collected from transgenic and non-transgenic cotton and analyzed for various fiber quality parameters. Data analyses revealed that fiber quality parameters were improved in the cotton plants engineered with the Δ*Gh_A07G1537* gene. Fiber length was found to be improved by 4.4% (31.5mm), fiber strength 3.0% (33.2g/tex), uniformity index 4.2% and micronaire value by 6.9%. Hence, overall parameters of fiber quality were improved in the transgenic cotton plants as compared with those of the non-transgenic plants. However, boll weight was found to be reduced by 12% (see Figure 10).

## Discussion

Cotton is one of the most important and integrated economic crops worldwide that produces high-quality natural fiber, which is being exploited by traditional breeding and molecular genomics methods for improved fiber quality and yield. Molecular approaches are more reliable and sophisticated than traditional breeding to get the desired characteristics of fiber quality and yield (Wilkins and Arpat, 2005). The improvement in cotton fiber quality through genetic modifications is a marked economic development in a short time (Ahmed et al., 2018b). *Agrobacterium*-mediated transformation directly correlates with improvement in crop characteristics (Zhang, 2013). Therefore, the current study was performed with *Agrobacterium*-mediated transformation.

Different plant expression vectors are used for plant transformation under efficient promoters. Promoter is a specified Segment of DNA that initiates a transcription of a specific gene (Yaqoob et al., 2020). Promoters are selected or designed based on the expression of the required gene. Some of the promoters are constitutive whereas others are tissue specific. CaMV35S is most commonly used as constitutive promoter that expresses in all parts of the plant at different development stages. This promoter is used to study the transient and stable expression of the gene. For example, CaMV35S promoter is widely adopted and reported efficient transformation of foreign gene into the cotton plant. In a recent study of sucrose synthase (*SuS*) gene transformation in cotton, CaMV35S promoter is used and resulted in significant improvement in fiber quality (Ahmed et al., 2020). This has been proved through various studies that revealed the relative expression of the tissue specific and constitutive promoters. The level of expression of the gene in cotton plant indicates the difference between constitutive and tissue specific promoter (Yaqoob et al., 2020). The introduction of gene for overexpression in cotton using CaMV35S promoter causes visible differences in plant phenotypes such as plant height, boll number, boll weight, fiber strength and length (Li et al., 2015).

The current study is the continuity of the previous study conducted at The Institute of Cotton Research-CAAS, Anyang, Henan, China. Different CSSLs such as MBI7561, MBI7285, and MBI7747 were developed. The transcriptome and RNA-seq analysis revealed some potential genes involved in the development of fiber length at 20 DPA. It was identified that the gene *Gh_A07G1537* belongs to the ZFPs family and directly correlates with the primary wall biosynthesis (Li et al., 2021) and thus brought under experimentation for further functional validation.

In this proposed study, the potential gene *Gh_A07G1537* was isolated and amplified from the material MBI7747 and transformed into cotton variety CCRI24 through *Agrobacterium*-mediated transformation. Before its transformation, the gene was cloned for sequence analysis to compare the genotypic difference among MBI7747, CCRI45, and Hai1. It was found that MBI7747 showed an SNP and a complete Indel of 55bp than its parents CCRI45 and Hai1. This finding strengthens the previous hypothesis that this gene might contribute a significant addition to the improvement of fiber length just like other transgenes such as expansin (Bajwa et al., 2015), cellulose synthase (Arioli et al., 1998), sucrose synthase (Ahmed et al., 2020), and actin (Ahmed et al., 2018a).

The southern blot analysis and copy number were performed to confirm the integration of the gene (*Gh_A07G1537*) into the transgenic plants. Transgenic plants were integrated with two copy numbers and non-transgenic plants were integrated with a single copy number into their genome (see Figure 8). It was found that the expression level of the transgenic plants with two copy numbers was higher than that in the non-transgenic plants with a single copy number. The higher expression of the gene may be due to copy number in transgenic plants, gene positional effects, gene insertion effects, internal cell programming, and environmental factors (Southern, 1975). Similar results were reported by Cantsilieris et al. (2013).

Furthermore, the quantitative expression analysis was performed through the qRT-PCR for the differential expression of the gene. The Quantitative Real-time PCR for the analysis of transgenic and non-transgenic plants was performed which revealed a significant differential expression of the gene. The leaves of the three transgenic plant generations (T_0_, T_1_, and T_2_) each from the same cotton variety CCRI24 was taken and the variations in the relative expression of the gene in three transgenic plant generations were observed (see Figure 9). It was found that the relative overexpression of T_0_, T_1_, and T_2_ was 2.97-, 2.86-, and 2.92-folds higher than that of the non-transgenic plants. Similar results for cotton fiber quality improvement were presented by Yang et al. (2009), Corrêa et al. (2010), and Li et al. (2015). The overall difference of expression among the three generations was very low that may have been due to environmental stresses or human handling. However, it indicates stable transgene integration into the host genome.

A careful examination of the cotton fiber samples from the transgenic and non-transgenic cotton lines showed that in the transgenic plants, fiber length was improved by 4.4% (31.5mm), fiber strength 3.0% (33.2g/tex), uniformity index 4.2%, and micronaire value 2.4% compared to those in the non-transgenic plants, whereas boll weight was reduced to 12%. Interestingly, the same cotton variety was cultivated in Anyang, China and it showed fewer promising results. Comparing with the results of non-transgenic CCRI24 cultivated in China and Pakistan, the cotton fiber quality parameters observed in Pakistan were even considerable than recorded in China.

Henceforth, the results of the current study revealed that overexpression of the Δ*Gh_A07G1537* gene contributed significantly to the improvement of fiber length in cotton. Hence, overexpression of Δ*Gh_A07G1537* may pave the way to the development of future cotton crops with improved and desired fiber quality.

Furthermore, we anticipate that the learnings gained from this study would be valuable in the future to exploit the gene Δ*Gh_A07G1537* through gene-editing technology for the improvement of fiber quality.

## Conclusion

The present study revealed that overexpression of the Δ*Gh_A07G1537* gene in cotton has led to improved fiber quality parameters. The molecular analysis showed that the expression of the gene in transgenic plants was higher than that in non-transgenic plants. Further, the expression of the transgene in T_0_, T_1_, and T_2_ generations indicated its stable integration into the host genome. This overexpression of the genes ultimately resulted in the improvement of fiber length, since a significant increase in the fiber length and fiber strength may be of great value for the textile industry.

## Data Availability Statement

The original contributions presented in the study are included in the article/supplementary material, further inquiries can be directed to the corresponding authors.

## Author Contributions

AR wrote the initial draft of the manuscript. MZ, AH, GQ, and XD made all necessary corrections and carried out final editing of the manuscript. PL and YS provided the material. AA provided the lab space. AL provided technical support. MR, HS, and WG proofread the manuscript. Final approval for publication was given by the group leader at The Institute of Cotton Research. All authors contributed to the article and approved the submitted version.

## Funding

This work was funded by China Agriculture Research System of MOF and MARA, the Natural Science Foundation of China (31471538 and 31371668), the National Key R&D Program of China (2017YFD0101603-11 and 2016YFD0100500), the Agricultural Science and Technology Innovation Program for CAAS (CAAS-ASTIP-ICRCAAS), the National High Technology Research and Development Program of China (2012AA101108 and 2009AA101104), and the Central Level of the Scientific Research Institutes for Basic R&D Special Fund Business (1610162014008).

## Conflict of Interest

AA is an employee of FB Genetics Four Brothers Group. FB Genetics is a non-profit and research-based unit of Four Brothers Group.

The remaining authors declare that the research was conducted in the absence of noncommercial and financial relationships that could be constructed as a potential conflict of interest.

## Publisher’s Note

All claims expressed in this article are solely those of the authors and do not necessarily represent those of their affiliated organizations, or those of the publisher, the editors and the reviewers. Any product that may be evaluated in this article, or claim that may be made by its manufacturer, is not guaranteed or endorsed by the publisher.

## References

[ref1] Ahmed M. Iqbal A. Latif A. Sarwar M. B. Wang X. Rao A. Q.. (2020). Overexpression of a sucrose synthase gene indirectly improves cotton fiber quality through sucrose cleavage. Front. Plant Sci. 11:476251. doi: 10.3389/fpls.2020.476251, PMID: 33281834 PMC7688987

[ref2] Ahmed M. Shahid A. A. Akhtar S. Latif A. ud Din S. Fanglu M.. (2018a). Sucrose synthase genes: a way forward for cotton fiber improvement. Biologia 73, 703–713. doi: 10.2478/s11756-018-0078-6

[ref3] Ahmed M. Shahid A. A. Din S. U. Akhtar S. Ahad A. Rao A. Q.. (2018b). An overview of genetic and hormonal control of cotton fiber development. Pak. J. Bot. 50, 433–443.

[ref4] Arioli T. Peng L. Betzner A. S. Burn J. Wittke W. Herth W.. (1998). Molecular analysis of cellulose biosynthesis in *Arabidopsis*. Science 279, 717–720. doi: 10.1126/science.279.5351.717, PMID: 9445479

[ref5] Bajwa K. S. Shahid A. A. Rao A. Q. Bashir A. Aftab A. Husnain T. (2015). Stable transformation and expression of GhEXPA8 fiber expansin gene to improve fiber length and micronaire value in cotton. Front. Plant Sci. 6:838. doi: 10.3389/fpls.2015.00838, PMID: 26583018 PMC4628126

[ref6] Cantsilieris S. Baird P. N. White S. J. J. G. (2013). Molecular methods for genotyping complex copy number polymorphisms. Genomics 101, 86–93. doi: 10.1016/j.ygeno.2012.10.004, PMID: 23123317

[ref7] Corrêa A. C. de Morais Teixeira E. Pessan L. A. Mattoso L. H. C. J. C. (2010). Cellulose nanofibers from curaua fibers. Cellulose 17, 1183–1192. doi: 10.1007/s10570-010-9453-3

[ref8] Eshed Y. Zamir D. J. E. (1994). A genomic library of *Lycopersicon pennellii* in *L. esculentum*: a tool for fine mapping of genes. Euphytica 79, 175–179. doi: 10.1007/BF00022516

[ref9] Guo K. Du X. Tu L. Tang W. Wang P. Wang M.. (2016). Fibre elongation requires normal redox homeostasis modulated by cytosolic ascorbate peroxidase in cotton (*Gossypium hirsutum*). J. Exp. Bot. 67, 3289–3301. doi: 10.1093/jxb/erw146, PMID: 27091877 PMC4892722

[ref10] Guo Y. H. Yu Y. P. Wang D. Wu C. A. Yang G. D. Huang J. G.. (2009). GhZFP1, a novel CCCH-type zinc finger protein from cotton, enhances salt stress tolerance and fungal disease resistance in transgenic tobacco by interacting with GZIRD21A and GZIPR5. New Phytol. 183, 62–75. doi: 10.1111/j.1469-8137.2009.02838.x 19402879

[ref11] Hall T. M. (2005). Multiple modes of RNA recognition by zinc finger proteins. Curr. Opin. Struct. Biol. 15, 367–373. doi: 10.1016/j.sbi.2005.04.004, PMID: 15963892

[ref12] Kanat S. Abbasi S. A. Peerzada M. H. Atilgan T. J. I. T. (2018). SWOT analysis of Pakistan's textile and clothing industry. Text 69, 502–510. doi: 10.35530/IT.069.06.1488

[ref13] Kong Z. Li M. Yang W. Xu W. Xue Y. (2006). A novel nuclear-localized CCCH-type zinc finger protein, OsDOS, is involved in delaying leaf senescence in rice. Plant Physiol. 141, 1376–1388. doi: 10.1104/pp.106.082941, PMID: 16778011 PMC1533915

[ref14] Li X.-B. Cai L. Cheng N.-H. Liu J.-W. (2002). Molecular characterization of the cotton GhTUB1 gene that is preferentially expressed in fiber. Plant Physiol. 130, 666–674. doi: 10.1104/pp.005538, PMID: 12376634 PMC166596

[ref15] Li P. Lu Q. Xiao X. Yang R. Duan X. J. P. (2021). Dynamic expression analysis and introgressive gene identification of fiber length using chromosome segment substitution lines from *G. hirsutum* × *G. barbadense*. Phyton 90:129. doi: 10.32604/phyton.2021.012928

[ref16] Li Z. Thomas T. L. (1998). PEI1, an embryo-specific zinc finger protein gene required for heart-stage embryo formation in *Arabidopsis*. Plant Cell 10, 383–398. doi: 10.1105/tpc.10.3.383, PMID: 9501112 PMC143998

[ref17] Li B. Yang Y. Hu W. R. Li X. D. Cao J. Q. Fan L. J. P. B. (2015). Overexpression of Gh UGP 1 in upland cotton improves fibre quality and reduces fibre sugar content. Plant Breed. 134, 197–202. doi: 10.1111/pbr.12247

[ref18] Liu Q. Qin J. Li T. Liu E. Fan D. Edzesi W. M.. (2015). Fine mapping and candidate gene analysis of qSTL3, a stigma length-conditioning locus in rice (*Oryza sativa* L.). PLoS One 10:e0127938. doi: 10.1371/journal.pone.0127938 26030903 PMC4452489

[ref19] Liu C. Zhang T. (2017). Expansion and stress responses of the AP2/EREBP superfamily in cotton. BMC Genomics 18:118. doi: 10.1186/s12864-017-3517-9, PMID: 28143399 PMC5282909

[ref20] Manik N. Ravikesavan R. J. B. (2009). Emerging trends in enhancement of cotton fiber productivity and quality using functional genomics tools. Biotechnol. Mol. Biol. Rev. 4, 11–28.

[ref21] Moore M. Ullman C. (2003). Recent developments in the engineering of zinc finger proteins. Brief. Funct. Genom. Proteomic. 1, 342–355. doi: 10.1093/bfgp/1.4.342, PMID: 15239882

[ref22] Pu L. Li Q. Fan X. Yang W. Xue Y. J. G. (2008). The R2R3 MYB transcription factor GhMYB109 is required for cotton fiber development. Genetics 180, 811–820. doi: 10.1534/genetics.108.093070, PMID: 18780729 PMC2567382

[ref23] Rapp R. A. Haigler C. H. Flagel L. Hovav R. H. Udall J. A. Wendel J. F. (2010). Gene expression in developing fibres of upland cotton (*Gossypium hirsutum* L.) was massively altered by domestication. BMC Biol. 8:139. doi: 10.1186/1741-7007-8-139, PMID: 21078138 PMC2992495

[ref24] Seagull R. W. Oliveri V. Murphy K. Binder A. Kothari S. J. J. C. S. (2000). Cotton fiber growth and development 2. Changes in cell diameter and wall birefringence. J. Cotton Sci. 4, 97–104.

[ref25] Shi Y. Li W. Li A. Ge R. Zhang B. Li J.. (2015). Constructing a high-density linkage map for *Gossypium hirsutum* × *Gossypium barbadense* and identifying QTLs for lint percentage. J. Integr. Plant Biol. 57, 450–467. doi: 10.1111/jipb.12288, PMID: 25263268

[ref26] Southern E. M. (1975). Detection of specific sequences among DNA fragments separated by gel electrophoresis. J. Mol. Biol. 98, 503–517. doi: 10.1016/S0022-2836(75)80083-0, PMID: 1195397

[ref27] Stege J. T. Guan X. Ho T. Beachy R. N. Barbas C. F. III. (2002). Controlling gene expression in plants using synthetic zinc finger transcription factors. Plant J. 32, 1077–1086. doi: 10.1046/j.1365-313x.2002.01492.x, PMID: 12492848

[ref28] Stelly D. Saha S. Raska D. Jenkins J. J. C. S. (2005). Registration of 17 upland (*Gossypium hirsutum*) cotton germplasm lines disomic for different *G. barbadense* chromosome or arm substitutions. Crop Sci. 45:2663. doi: 10.2135/cropsci2004.0642

[ref29] Ulian E. Smith R. Gould J. McKnight T. (1988). Transformation of plants via the shoot apex. In Vitro Cell. Dev. Biol. 24, 951–954. doi: 10.1007/BF02623909

[ref30] Von Mark V. C. Dierig D. A. (2014). Industrial Crops: Breeding for Bioenergy and Bioproducts. New York, NY: Springer.

[ref31] Wang D. Guo Y. Wu C. Yang G. Li Y. Zheng C. (2008). Genome-wide analysis of CCCH zinc finger family in *Arabidopsis* and rice. BMC Genomics 9:44. doi: 10.1186/1471-2164-9-44, PMID: 18221561 PMC2267713

[ref32] Wang P. Zhang S. Qiao J. Sun Q. Shi Q. Cai C.. (2019). Functional analysis of the GbDWARF14 gene associated with branching development in cotton. PeerJ 7:e6901. doi: 10.7717/peerj.8300, PMID: 31143538 PMC6524629

[ref33] Wilkins T. A. Arpat A. B. (2005). The cotton fiber transcriptome. Physiol. Plant. 124, 295–300. doi: 10.1111/j.1399-3054.2005.00514.x

[ref34] Wu Y. Liu F. Yang D.-G. Li W. Zhou X.-J. Pei X.-Y.. (2018). Comparative chloroplast genomics of Gossypium species: insights into repeat sequence variations and phylogeny. Front. Plant Sci. 9:376. doi: 10.3389/fpls.2018.00376, PMID: 29619041 PMC5871733

[ref35] Xiao G. Zhao P. Zhang Y. J. (2019). A pivotal role of hormones in regulating cotton fiber development. Front. Plant Sci. 10:87. doi: 10.3389/fpls.2019.00087, PMID: 30838005 PMC6382683

[ref36] Yang Z. Li J. Li A. Zhang B. Liu G. Li J.. (2009). Developing chromosome segment substitution lines (CSSLs) in cotton (*Gossypium*) using advanced backcross and MAS. Mol. Plant Breed. 7, 233–241.

[ref37] Yaqoob A. Ali Shahid A. Salisu I. B. Shakoor S. Usmaan M. Shad M.. (2020). Comparative analysis of constitutive and fiber-specific promoters under the expression pattern of expansin gene in transgenic cotton. PLoS One 15:e0230519. doi: 10.1371/journal.pone.0230519, PMID: 32187234 PMC7080281

[ref38] Zhang B. (2013). Agrobacterium-mediated transformation of cotton. Methods Mol. Biol. 958, 31–45. doi: 10.1007/978-1-62703-212-4_3, PMID: 23143481

[ref39] Zhang J. Percy R. G. McCarty J. C. J. E. (2014). Introgression genetics and breeding between upland and Pima cotton: a review. Euphytica 198, 1–12. doi: 10.1007/s10681-014-1094-4

